# *PIK3CA* mutation enrichment and quantitation from blood and tissue

**DOI:** 10.1038/s41598-020-74086-w

**Published:** 2020-10-13

**Authors:** Ieva Keraite, Virginia Alvarez-Garcia, Isaac Garcia-Murillas, Matthew Beaney, Nicholas C. Turner, Clare Bartos, Olga Oikonomidou, Maïwenn Kersaudy-Kerhoas, Nicholas R. Leslie

**Affiliations:** 1 Institute of Biological Chemistry, Biophysics and Bioengineering, Heriot-Watt University, Edinburgh, EH14 4AS UK; 2 Infection Medicine, Edinburgh Medical School, College of Medicine and Veterinary Medicine, The University of Edinburgh, Edinburgh, EH164SB UK; 3 Edinburgh Cancer Research Centre, University of Edinburgh, Crewe Road South, Edinburgh, EH4 2XR UK; 4 The Breast Cancer Now Research Centre, The Institute of Cancer Research, London, SW3 6JB UK; 5 Breast Unit, Royal Marsden Hospital, Fulham Road, London, SW3 6JJ UK; 6 Edinburgh Cancer Centre, Western General Hospital, Crewe Road South, Edinburgh, EH4 2XU UK; 7 Institute of Biological Chemistry, Biophysics and Bioengineering, School of Engineering and Physical Sciences, Heriot-Watt University, Edinburgh, EH14 4AS UK

**Keywords:** Biological techniques, Cancer, Genetics, Molecular biology, Diseases, Health care, Medical research, Molecular medicine, Oncology, Signs and symptoms

## Abstract

*PIK3CA* is one of the two most frequently mutated genes in breast cancers, occurring in 30–40% of cases. Four frequent ‘hotspot’ *PIK3CA* mutations (E542K, E545K, H1047R and H1047L) account for 80–90% of all *PIK3CA *mutations in human malignancies and represent predictive biomarkers. Here we describe a *PIK3CA* mutation specific nuclease-based enrichment assay, which combined with a low-cost real-time qPCR detection method, enhances assay detection sensitivity from 5% for E542K and 10% for E545K to 0.6%, and from 5% for H1047R to 0.3%. Moreover, we present a novel flexible prediction method to calculate initial mutant allele frequency in tissue biopsy and blood samples with low mutant fraction. These advancements demonstrated a quick, accurate and simple detection and quantitation of *PIK3CA* mutations in two breast cancer cohorts (first cohort n = 22, second cohort n = 25). Hence this simple, versatile and informative workflow could be applicable for routine diagnostic testing where quantitative results are essential, e.g. disease monitoring subject to validation in a substantial future study.

## Introduction

Breast cancer is the most frequently diagnosed cancer in women and it is also the leading cause of female cancer-related deaths worldwide despite the extensive progress that has been made during the last few decades regarding early detection and screening of patients and improved treatment methods^1^. Generally, therapeutic decisions are based on tumour histology and receptor status: ER (Estrogen Receptor), PR (Progesterone Receptor) and ERBB2, also known as HER2 (Human Epidermal Growth Factor Receptor 2), in tumour tissue biopsies. Increased understanding of tumour biology and number of available targeted therapies provide opportunities for better treatment, but require the development of new biomarkers with reliable, less invasive, and low-cost technologies to quantify them.

Phosphoinositide 3-kinase (PI3K) enzyme alterations have been recognised as one of the most frequent oncogenic drivers in many cancer types^2^. *PIK3CA,* an oncogene that encodes the p110α catalytic component of PI3K, is one of the most frequently mutated genes in human cancer and in particular breast cancer^3–5^. Point mutations in *PIK3CA*, which activate the PI3K enzyme and signalling pathway, are observed in 20–40% of breast cancer cases. More than 80% of these mutations are clustered in the sequences encoding the helical (c.1624G > A, p.E542K, and c.1633G > A, p.E545K) and kinase (c.3140A > G, p.H1047R, and c.3140A > T, p.H1047L) domains of the PI3K p110α protein^2,6^.

Substantial investment and research has succeeded in producing a range of small molecule drugs targeting PI3K, which have undergone clinical trials in cancer patients. Such trials have recently resulted in the FDA approval of Alpelisib/BYL719, a PI3K p110α selective inhibitor for advanced breast cancers which are hormone receptor positive, HER2 negative and *PIK3CA* mutant^7^. This and other clinical circumstances^8–15^ identify *PIK3CA* status as an important biomarker with both predictive and prognostic value, and motivate the development of reliable, facile, economic tests for these mutations.

In recent years, blood-based biomarkers, especially circulating cell-free DNA (cfDNA), have emerged as an alternative less invasive test to tissue biopsies, with the advantage of an easier repeated sampling procedure. Circulating tumour DNA might also reflect tumour heterogeneity better as it is thought to originate from distinct clones within a primary tumour as well as distant lesions if present. Therefore, cfDNA analysis provides the ability non-invasively to detect genomic alterations, including driver mutations, which is pivotal to track the development and recurrence of the disease, to predict resistance to therapies and to make decision on treatment^16–19^.

Current sample preparation techniques and molecular detection methods still face challenges, such as low yield of nucleic acid and lack of analytical sensitivity, robustly to detect mutations at low abundance in a background of high wild type DNA^20–23^. There are several strategies for improved selective detection of mutant alleles. They involve PCR-based assays such as COLD-PCR^24^, LNA-PCR prior to sequencing^25^, as well as thermal-electrophoretic separation SCODA^26^, DNA probes for hybrid capture^27^, restriction enzyme-based assays like dCas9^28^ and NaME-PrO^29–31^. Nuclease-based techniques appear to be more robust and have much better multiplexing capabilities as they are less technically demanding and do not require extensive optimisation as PCR-based methods.

The nuclease-assisted minor-allele enrichment assay with overlapping probes (NaME-PrO), recently developed by Makrigiorgos and collaborators^29^ is practical and cost-effective, and allows detection of multiple very low abundance mutations that could have relevance in the clinic. In this work, enrichment was shown to improve *PIK3CA* mutant allele detection by SYBR Green real-time qPCR^32,33^ and multiplex dPCR in a range of clinical samples. Although, digital PCR is an emerging sensitive approach for the detection of rare mutant alleles with absolute quantification, in many laboratories such instruments are not available. Not only instrumentation but the cost of reagents and consumables of our approach is much lower than dPCR or other qPCR techniques, such as TaqMan probe based qPCR. Therefore our method provides a simple, robust and low cost solution with quick turnaround time, which achieves comparable sensitivity to other qPCR approaches, to detect clinically relevant mutations at any laboratory with qPCR platform. Despite the advantages of capturing low allelic-fraction mutations, predicting the precise abundance of potential driver mutations in the original sample has not been demonstrated, which is important in disease management for patient follow-up and clinical decision making. Drawing on advances in nuclease mediated enrichment, we describe here an enrichment method for the 4 most common *PIK3CA* mutant alleles, and their detection with PCR-based techniques both from breast cancer tissue and from cfDNA samples. We also introduce and validate a novel prediction model to calculate the initial variant-allele fraction in the clinical samples prior to enrichment.

## Materials and methods

### Cell lines

The human T-47D and MCF-7 breast cancer cell lines (European Collection of Authenticated Cell Cultures) were used in experiments. Cells were maintained in Dulbecco’s Modified Eagle Medium (Life Technologies) containing 10% foetal bovine serum (Invitrogen) and 100 units/ml penicillin–streptomycin (Sigma-Aldrich). Cells were cultured at 37 °C under a 5% CO2 atmosphere.

CAL-51 and EFM-19 cell line DNAs were purchased from Leibniz Institute DSMZ-German Collection of Microorganisms and Cell Cultures GmbH. Genomic DNA from these cell lines was used for method optimisation as described below.

Cell lines were selected based on mutations in the *PIK3CA* gene: T-47D cells are heterozygous for H1047R mutation, MCF-7—heterozygous for E545K mutation, CAL-51—heterozygous for E542K mutation, and EFM-19 cells carry a heterozygous H1047L mutation. *PIK3CA* copy number and variant allele fraction (VAF) apparent in each cell line has been confirmed in the canSAR v4.0 tool and experimentally (data not shown): T-47D cell line carries 6 copies (VAF ~ 50%), MCF-7—3 copies (VAF ~ 30%), CAL-51—2 copies (VAF ~ 50%), and EFM-19—4 copies (VAF ~ 50%). *PIK3CA* copy number and mutant allele frequency in each cell line were considered in calculations in order to prepare lower mutant allele fraction controls by dilution with wild type human genomic DNA.

### Clinical samples

From a set of 22 sample pairs previously collected by the Oncology team of the Western General Hospital in Edinburgh, UK, five matching fresh frozen core biopsies and plasma sample pairs from female patients with a newly diagnosed breast cancer (ER/PR—positive, HER2 negative) positive for one of the *PIK3CA* mutations (E542K, E545K, H1047R) were chosen (Table S1A). The study was approved by the Scotland Research Ethics Committee (15/ES/0094) and was previously published^32^. Informed consent was obtained from all individual participants included in the study. The *PIK3CA* status of the tissue biopsy samples was verified by Next-Generation Sequencing analysis using the Illumina Tru-Seq Amplicon Cancer Panel. Genomic DNA extracted from frozen core biopsies was processed at the Edinburgh Genomics facility according to the manufacturer’s instructions and sequenced on an Illumina MiSeq using a 250 base paired-end sequencing strategy (version 2 chemistry) to a depth of 1000× with > 99.8% mapped reads in all samples. Blood samples were collected in EDTA tubes and processed within 4 h following venepuncture. Plasma was isolated by double centrifugation for 10 min each time at 1600 g and 12,100 g respectively.

A second set of 25 samples, FFPE tumour tissue from breast cancer patients, was independently collected at the Institute of Cancer Research, London, UK (Table S1B). Samples were collected from a consecutive series of patients with metastatic breast cancer treated at the Royal Marsden Hospital between 2010 and 2012. Research was approved by the Royal Marsden Hospital Research Ethics Committee (REC Ref No: 10/H0805/50).

### DNA extraction and quantification

DNA was extracted from cells in culture using the DNeasy Blood and Tissue Kit (Qiagen) following manufacturer’s protocol in an elution volume of 200 µl. Genomic DNA was extracted from 25 mg of fresh frozen core biopsies using the QIAamp DNA mini kit (Qiagen) in an elution volume of 100 µl. Circulating cell-free DNA was extracted from an average of 5 ml of plasma using the QIAamp Circulating Nucleic Acid Kit (Qiagen) in an elution volume of 20 µl. Concentration of genomic DNA from cells and frozen core biopsies was measured using the Qubit V3 (Invitrogen) and HS DNA kit (Invitrogen). Circulating cell-free DNA was quantified by qPCR against a standard curve of known DNA concentrations. Quantitative PCR was performed using 2× Power SYBR Green PCR Master Mix (Thermo Fisher Scientific) to amplifying LINE-1 (long interspersed nuclear elements) target of 90 bp. The total reaction volume was 12.5 μl with a final concentration of each primer of 200 μM (Fw 5′-TGCCGCAATAAACATACGTG-3′, Rv 5′-GACCCAGCCATCCCATTAC-3′) and 1 μl cfDNA elution. Thermal cycling conditions involved a 10 min cycle at 95 °C followed by 40 cycles with 15 s at 95 °C and 60 s at 60 °C. Samples were amplified in triplicates using StepOnePlus Real-Time PCR System (Applied Biosystems). A melting curve was performed as a control measure for nonspecific amplification. The standard curve for absolute quantification of cfDNA was created with commercially available human genomic DNA (Bioline) with a linear range over 5 orders of magnitude (R^2^ > 0.98).

Extracted DNA was aliquoted and stored at − 20 °C until use. DNA from FFPE samples was isolated using the QIAamp DNA FFPE Tissue Kit (Qiagen) as per manufacturer’s instructions and *PIK3CA* status determined by ddPCR on a Bio-Rad QX-200 digital PCR as previously described^34^.

All experiments were performed in accordance with MIQE guidelines and regulations^35,36^.

### PCR pre-amplification

FFPE sample DNA yield and mutant allele abundance were identified after initial dPCR analysis. FFPE samples of low DNA yield (< 10 ng) and low mutant allele abundance (< 15%), and cfDNA samples were pre-amplified before enrichment. PCR reactions were performed on TC-512 thermal cycler (Techne) using the Phusion Hot Start II High-Fidelity PCR Master Mix (Thermo Fisher Scientific). All primers were designed with the IDT OligoAnalyzer tool (exon 9 and exon 20) and were purchased from Eurofins Genomics (Fig. S2C). Reactions had a total volume of 12.5 μl containing 6.25 μl 1× Phusion HS II HF Master mix, 500 nM of forward and reverse primer each, 1–3 ng DNA template and molecular grade water to the final volume. A two-step PCR consisting of an initial denaturation step at 98 °C for 30 s, followed by 15 cycles of 15 s denaturation at 98 °C and 1 min annealing at 60 °C was conducted. Samples were held at 4 °C before amplicon clean-up, which was performed using Beckman Coulter Agencourt AMPure XP SPRI magnetic beads following manufacturer’s instructions. PCR products were eluted in 20 μl nuclease-free water and stored at − 20 °C until use.

### Nuclease-based enrichment assay

Nuclease-assisted minor-allele enrichment with probe-overlap specific to wild type DNA was designed for the enrichment of a set of *PIK3CA* mutations (E542K, E545K, H1047L, H1047R). Overlapping probes were designed with IDT OligoAnalyzer tool according to the assay requirements (Table S2A)^29^. DNA from cell lines harbouring the desired mutation was mixed with wild type DNA to obtain a decreasing mutational abundance from in the range of 12.5–0.025%. Wild Type human genomic DNA (Bioline) was used as control. Each reaction contained 1 µl of 10× DSN buffer, top and bottom strand probes (20–50 nM final concentration), 5 µl of cell line or tissue DNA (20 ng/µl) or amplified DNA, and DNAse-free water up to a volume of 10 µl. Samples were denatured on TC-512 (Techne) thermal cycler at 98 °C for 2 min. The temperature was then reduced to 67 °C and 0.2 units of Duplex Specific Nuclease (DSN) (Evrogen) were added into the mixture followed by 20 min incubation at 67 °C and 2 min at 95 °C for DSN inactivation. No-DSN controls were run in parallel in all reactions. No sample purification was performed after nuclease trea^tm^ent with samples stored at -20 °C until further use.

### qPCR for the detection of PIK3CA mutations

Allele-specific real-time quantitative PCR for *PIK3CA* H1047R and E545K mutations has been described previously^32^ and was combined with the enrichment assay^29^. Additional qPCR assays were developed to detect E542K and H1047L mutations for this study. qPCR primers and blocking oligonucleotides were purchased from Eurofins Genomics (Table S2B). Reactions targeting the mutant sequence were performed in a final reaction volume of 12.5 µl consisting of 6.25 µl of 2× Power SYBR Green master mix (Life Technologies), 1.25 µl of each forward and reverse primers (final concentration 100 nM) and blocking oligonucleotide (final concentration 200 nM), and 2.5 µl of DSN enriched DNA sample or untreated control. Internal control reactions were prepared following the same protocol, however 1.25 µl of nucleic acid-free water was used instead of the blocking primer. Cycling conditions on Agilent Mx3005P qPCR system were 95 °C for 10 min, followed by 40 cycles of 95 °C for 15 s and 60 °C for 60 s. Melting curve analysis was performed to confirm single, specific amplification product. qPCR data was analysed by the comparative ΔΔCt method to calculate change in fold amplification relative to wild type human genomic DNA (Bioline). Tested samples were considered to carry the mutation when the relative amplification of the mutant allele was statistically significant in comparison to a wild type control. DNA from *PIK3CA* mutation positive cell lines was diluted down in wild type DNA reducing the ratio of mutant to wild type DNA. Genomic DNA derived from CAL-51, MCF-7 and T-47D cell lines was used as a positive control to validate E542K, E545K, and H1047R mutation detection respectively on DSN enriched samples. All reactions were carried out in duplicates due to the volume restriction in the enrichment assay and repeated at least three times.

### Digital PCR

Crystal digital PCR was performed on the Naica system (Stilla Technologies, France). All assays were developed and optimised to work with primers and probes designed with the IDT OligoAnalyser tool (Table S3). Firstly, a dPCR duplex assay was designed to detect the H1047R mutation. Furthermore, two triplex assays were developed and optimised for exon 9 (E542K and E545K mutations and WT) and exon 20 (H1047L and H1047R and WT) detection. All dPCR reactions were prepared in a total volume of 25 µl containing 5× PerFecTa Multiplex qPCR ToughMix (Quanta Biosciences), high purity grade fluorescein sodium salt (VWR) at a final concentration of 100 nM, 1 μl of 25× primer and probe multiplex mix and 1–10 μl of DNA template. Final probe concentration in duplex and triplex assays was 250 nM for each probe, while final primer concentration in duplex assay was 1 µM per primer and 500 nM per primer in triplex assays. Cycling conditions on the Naica Geode (Stilla Technologies, France) were 95 °C for 10 min, followed by 45 cycles of 95 °C for 30 s and 64 °C for 60 s for *PIK3CA* H1047R and exon 20 assays, or 63 °C for 60 s for *PIK3CA* exon 9 assay. Sapphire chips containing the 2D crystals with the 15,000 to 22,000 droplets generated were imaged using the Naica Prism3 reader and fluorescent data were analysed using Crystal Miner software (Stilla Technologies). Negative and positive droplets were discriminated using manual thresholding.

### Statistical analysis

Statistical analysis was performed using GraphPad Prism 7 (GraphPad Software Inc). Statistical significance between the tested groups was determined by paired parametric Student t-test.

## Results

### Enrichment assay performance in human cell lines followed by qPCR detection

**Figure 1:**
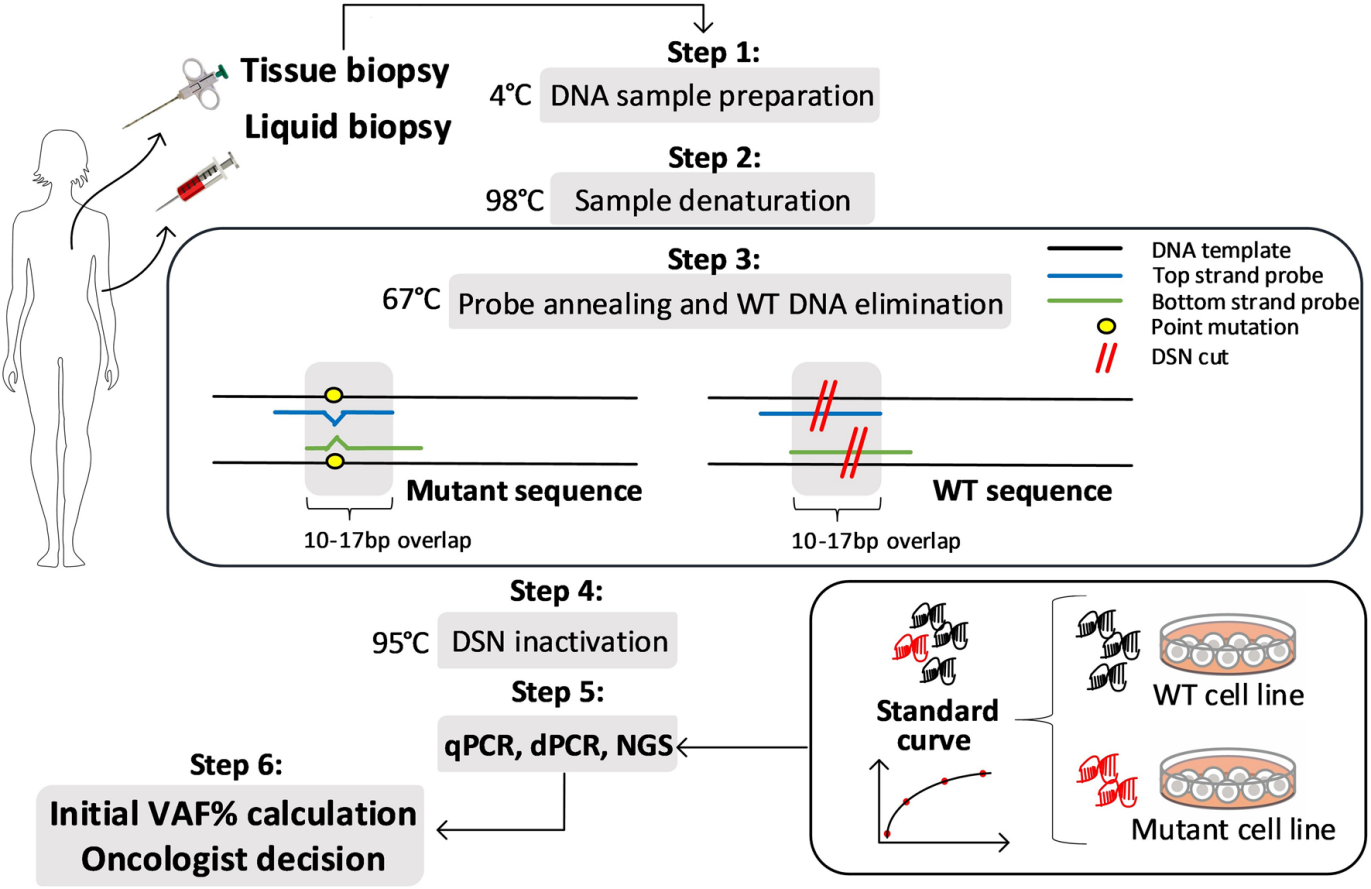
Workflow of nuclease-based mutant allele enrichment assay and initial VAF prediction. The enrichment protocol is based on duplex specific nuclease (DSN) preferentially degrading perfectly annealed double stranded DNA (dsDNA). DNA sample preparation introducing oligonucleotide probes which overlap the mutation location and are specific to the wild type (WT) sequence (step 1), is followed by sample denaturation (step 2), probe annealing to target DNA sequences and DSN digestion at perfectly annealed WT sequences (step 3) and nuclease inactivation at the end (step 4), thus enriching for mutant DNA sequences. After sample analysis with the user’s preferred method (step 5) initial VAF is calculated from a standard curve of known VAF DNA samples (step 6) and can be used to follow-up patient progression in the clinical setting.

We aimed to develop an enrichment protocol based on the NaME-PrO technique^29^ that could be used to efficiently enrich for low fractional abundance of several *PIK3CA* mutations (E542K, E545K, H1047R, and H1047L) from a high background of wild type DNA. Furthermore, we used data from standard samples with known fractions of mutant DNA accurately to predict the initial variant allele fraction in the test samples. The workflow consists of mutant sequence enrichment, based on overlapping probes that guide duplex specific nuclease (DSN) for wild type sequence elimination (Fig. 1).

In the initial phase of the study, which consisted of optimising nuclease enrichment approaches, mutant sequence enrichment was assessed in a following simple SYBR green based mutant allele specific qPCR and control reactions^32^. To evaluate the achievable mutant allele enrichment of *PIK3CA* mutations, we used genomic DNA from CAL-51, MCF-7, T-47D and EFM-19 cells known to carry the most common *PIK3CA* hotspot mutations—E542K, E545K, H1047R and H1047L respectively. MCF-7, T47D and EFM-19 cell lines are also known to have an amplification of the *PIK3CA* gene and which was considered when preparing cell line-derived genomic DNA to make decreasing mutant fraction samples of ~ 10, 5, 2.5, 1.25, 0.6, 0.3, and 0.15%. Two sets of overlapping probes to direct the nuclease to specific wild-type sequences and enable *PIK3CA* mutation enrichment were designed, one of which was specific to enrich both E542K and E545K mutations (exon 9), while the second set was specific to enrich H1047R and H1047L targets (exon 20) (Table S2A). These enrichment methods were applied to a total of 100 ng genomic DNA, comprising specified fractions of mutant and wild type DNA. Mutant allele-specific qPCR was then used to quantify the enrichment of each target mutation. The E542K specific qPCR assay with a wild type allele blocker had a limit of detection (LOD) of 5%. As previously reported^32^, the E545K specific qPCR assay had LOD of 10%. In combination with enrichment, both assays were significantly improved down to 0.6% (Fig. S1A,B). The *PIK3CA* H1047R specific qPCR assay^32^ had a reported LOD of 5%. Targeted enrichment of H1047R substantially enhanced the performance of the assay down to 0.3% (Fig. S1C). In order to detect the H1047L mutation using a simple qPCR assay, a new mutation-specific extending reverse primer was designed, while the wild type blocker and forward primer were retained. This assay had a LOD of 5% mutant fraction, which after enrichment was significantly reduced down to 0.3% (Fig. S1D). In brief, all four assays showed decreasing relative mutant allele amplification when mutant allele fraction was decreasing in both cases—enriched and untreated samples. However, relative amplification was significantly higher for the samples treated with DSN than those which did not undergo nuclease treatment.

### Linear regression model to predict VAF in low-abundance mutant clinical samples with qPCR detection method

**Figure 2:**
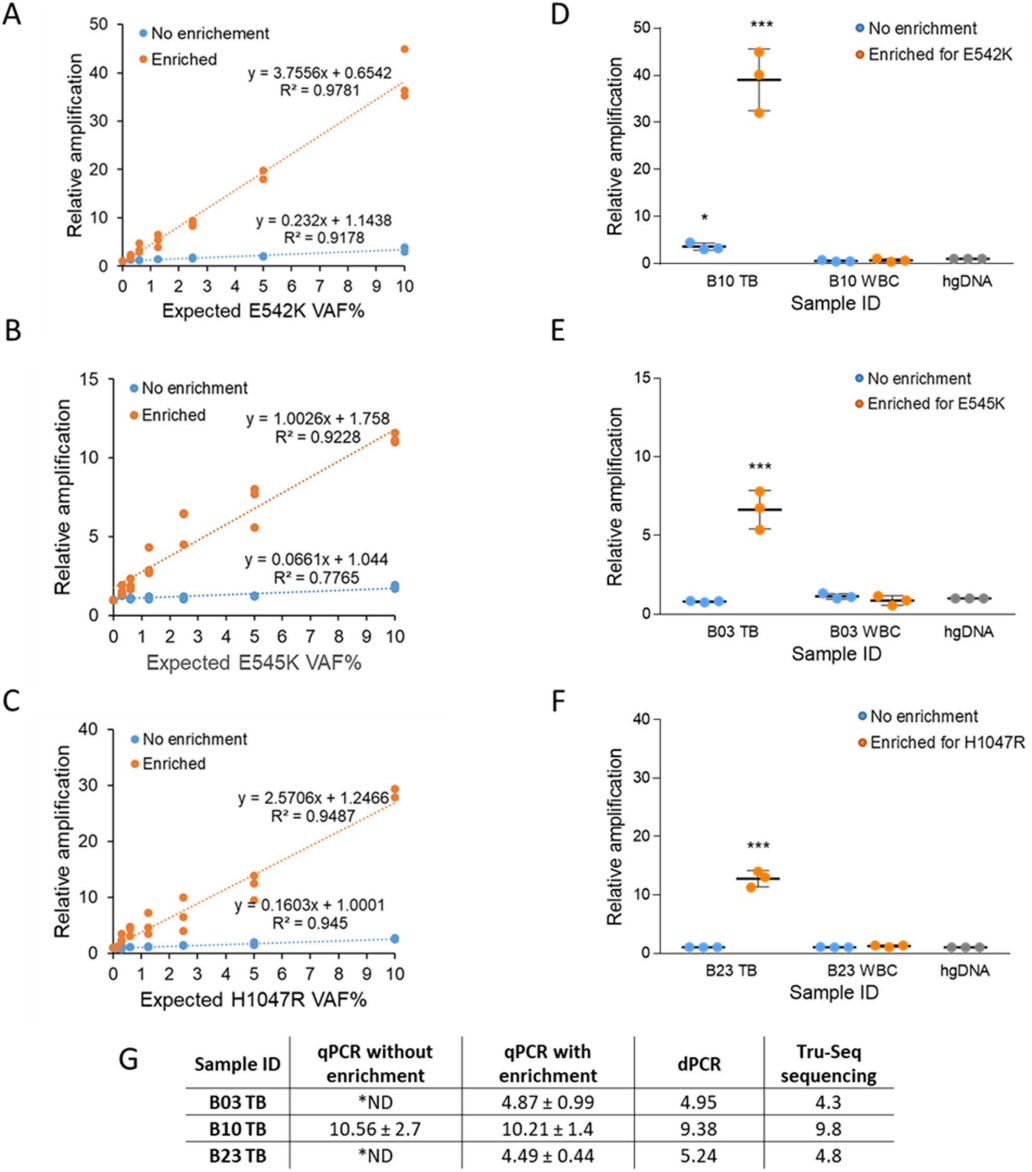
NaME-PrO wild type sequence elimination and mutant allele detection by SYBR Green qPCR. Genomic DNA from cell lines containing *PIK3CA* E542K (**A**), E545K (**B**), and H1047R (**C**) mutations were serially diluted in wild type DNA with decreasing mutation abundances. Mutation detection was performed in enriched samples and matched untreated controls by SYBR Green qPCR including a wild type blocking primer. A linear regression equation was estimated for data points for each mutation. *PIK3CA* mutation enrichment and detection assays (**D**) E542K, (**E**) E545K, (**F**) H1047R were applied to low mutation abundance tissue biopsies (TB) and white blood cell (WBC) control samples. Data was analysed by a ΔΔCt method, in which relative amplification was calculated relative to WT human genomic DNA (hgDNA), and shown as mean mutant fold amplification ± SD. Due to a limited enrichment reaction volume (10 µl), all qPCR points were obtained in duplicates in three independent experiments (n = 3). ***P < 0.001 compared with *PIK3CA* WT, *P < 0.05 compared with *PIK3CA* WT (Student’s t test). (**G**) Linear regression was applied to calculate the initial variant allele fraction for every tissue biopsy sample in qPCR experiments, and compared to dPCR and Tru-Seq panel sequencing results. *ND—no meaningful result, due to VAF < LOD.

We subsequently applied a linear regression model to estimate the original variant allele fraction in samples after enrichment for a given mutation. Data was collected from enrichment and detection experiments of *PIK3CA* mutations in samples of known VAF, and used to calculate the regression equation and coefficient of determination R^2^ for the specific assays detecting the E542K (Fig. 2A), E545K (Fig. 2B) H1047R (Fig. 2C) and H1047L (Fig. S1E) mutations. A strong correlation was observed in all models between expected variant allele fraction and relative amplification (R^2^ > 0.9) in enriched samples. This culminated with the establishment of a linear standard curve model to assess the initial E542K, E545K, H1047R, H1047L mutant allele fraction using a simple qPCR detection method.

We next validated our mutant allele prediction model with DNA samples obtained from frozen tissue biopsies of newly diagnosed breast cancer patients. In a previous study of 22 newly diagnosed breast cancer patients, positive *PIK3CA* E542K status was determined in one tissue biopsy sample, one patient was identified as positive for E545K, and three patients were confirmed to carry a H1047R mutation^32^. Therefore these five matching sample sets were chosen for further experiments.

Sequencing results (Fig. S2), verified by our dPCR method, showed that the E545K positive tissue biopsy B03 had a mutant allele fraction of < 5%, below the LOD of the mutation-specific qPCR assay without enrichment. The E542K positive tissue biopsy sample B10, also had a relatively low mutant allele fraction < 10%, hence it was included in further analysis. One of the three samples positive for H1047R, B23, was shown to harbour a low H1047R mutation abundance (< 5%) and could not be detected by the mutation specific qPCR method in a previous study^32^. Here, by combining the enrichment method for *PIK3CA* mutations and appropriate qPCR assays we were able to detect E542K in B10 (Fig. 2D), E545K in B03 (Fig. 2E) and H1047R in B23 (Fig. 2F) tissue biopsy samples with a significantly higher amplification signal in comparison to wild-type DNA. We then used these data and a linear regression modelling to predict the initial mutant allele fraction in each sample before enrichment (Fig. 2G). In each case, the initial variant allele fraction calculated by standard qPCR method, which for samples B03 and B23 could be predicted only if pre-enrichment was used, was very similar to the frequency determined by digital PCR and Tru-Seq panel sequencing (Fig. 2G).

### Enrichment assay validation in cell lines, blood and tissue samples by dPCR

To apply our established workflow of mutant allele enrichment and prediction of initial mutant allele fraction to circulating cell-free DNA (cfDNA) samples, we combined nuclease-based enrichment with a digital PCR detection method, providing both a better sensitivity and quantitative determination of template copy numbers.

**Figure 3:**
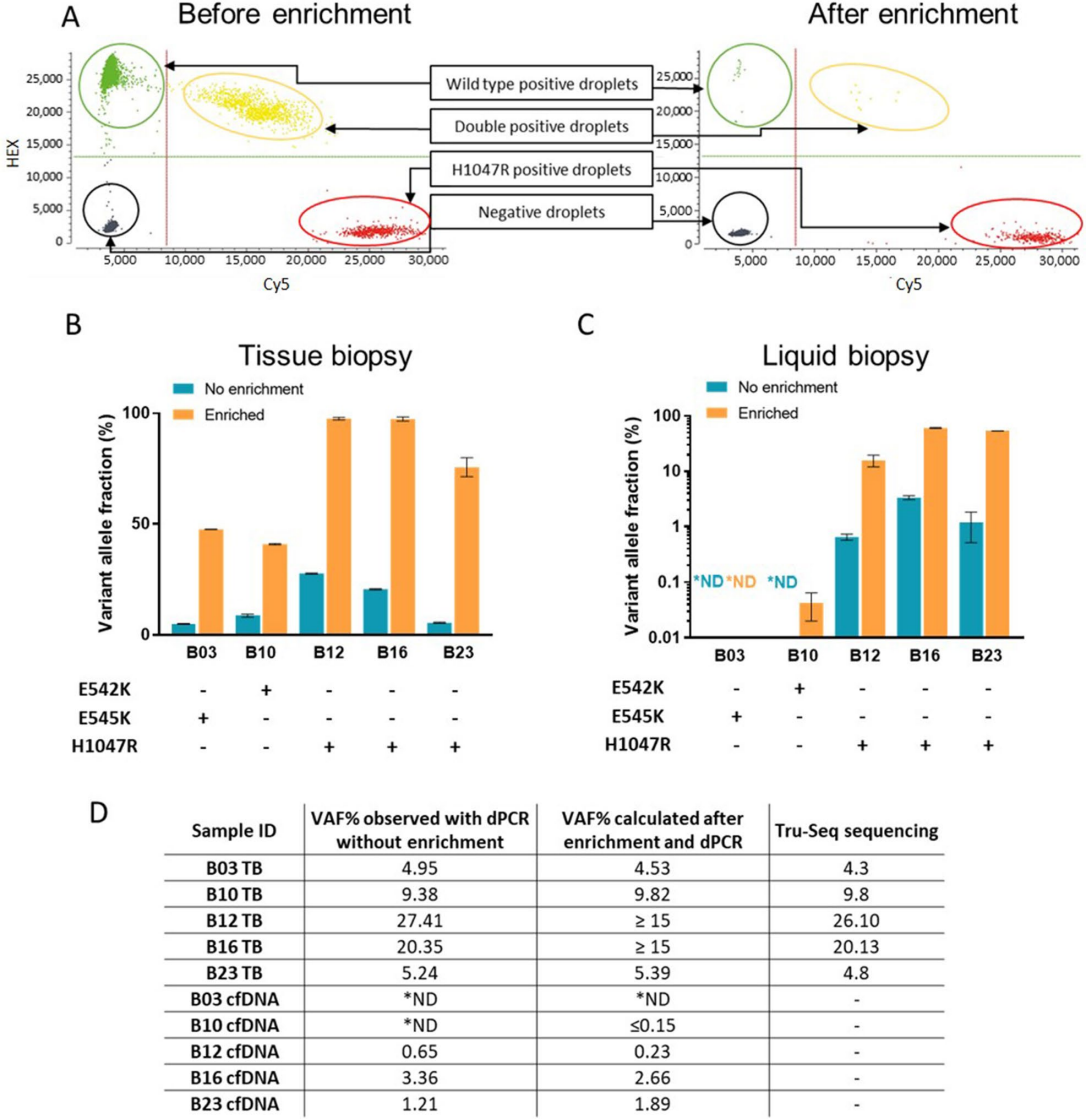
Validation of the *PIK3CA* mutation enrichment and detection methods with breast cancer tissue biopsy and cfDNA samples. (**A**) H1047R mutations were amplified from T-47D genomic DNA spiked into WT DNA at an allele fraction of approximately 10%. dPCR was used to evaluate mutation abundance before and after enrichment, with results of 8.07% and 95.7% respectively. Genomic DNA samples (**B**), obtained from frozen core biopsies of *PIK3CA* mutation positive breast cancer patients, and matched cfDNA samples from blood (**C**) were tested with dPCR E542K, E545K, H1047R mutant allele detection assays before and after enrichment. Due to low cfDNA yields in cfDNA extraction, samples were amplified prior to DSN nuclease-based enrichment assay. Clinical samples were tested only once due to lack of material for analysis. The results are presented with their Poisson-based 95% confidence intervals. (**D**) Logarithmic regression was applied to calculate each initial variant allele fraction for tissue biopsy samples and matching cfDNA samples after enrichment with nuclease-based assays and detection with dPCR. Calculated initial VAF% results were compared to dPCR prior to enrichment and Tru-Seq panel sequencing results (for tissue biopsy specimen only). *ND—not detected.

Firstly, digital PCR was used as a detection method to optimise *PIK3CA* mutation enrichment parameters in the cell line-derived genomic DNA samples diluted with wild type DNA to achieve a range of mutant fractions (12.5–0.025%). To illustrate the reduction of wild type DNA sequences, representative duplex dPCR 2D dot plots of the same sample (VAF 10%) before and after enrichment are shown in Fig. 3A. Significant enrichment of mutant allele were observed in all but the lowest mutant fractions (0.125 and 0.025%), where the number of mutant molecules is very small to endure the nuclease treatment. Therefore, amplification of the *PIK3CA* sequence surrounding the site of the H1047R mutation, was found beneficial for the samples of low mutant allele abundance (Fig. S3A). These observations were taken into consideration for further experiments with low VAF tissue and cfDNA where DNA input was limited.

To validate the utility of *PIK3CA* E542K, E545K and H1047R mutant allele enrichment for these mutations, frozen tissue biopsy genomic DNA and cfDNA extracted from plasma of breast cancer patients was used^32^. We first applied the duplex specific nuclease enrichment method, specific to exon 9 and exon 20–100 ng of tissue biopsy DNA (Fig. 3B). To evaluate the enrichment of target of interest we used in-house designed triplex dPCR assays for exon 9 (E542K, E545K and wild type) and exon 20 (H1047R, H1047L and wild type) (Table S3). Sample B03, positive for E545K, had an initial VAF of 4.95%, which after enrichment reached 47.5%. Sample B10 carried an E542K mutation at a VAF of 9.38%, which was increased to 41.1%. Samples B12 and B16, both positive for H1047R, showed a high initial mutant fraction of 20–30%, which was converted to 98% after enrichment. For a low H1047R mutant allele fraction sample B23 (VAF 5%), enrichment assay resulted in significant mutant allele fraction increase to 74.7%. We also performed *PIK3CA* mutant allele dPCR assays on matched patient blood samples before and after enrichment (Fig. 3C). Due to the very low circulating DNA amount and possibly low VAF, target amplification was done prior to enrichment. E545K mutation was not detected in cfDNA from patient B03 regardless of the enrichment. On the other hand, the enrichment assay enabled detection of E542K mutation in the patient B10 cfDNA sample, which was undetectable without conducting the enrichment assay. The other three patient-derived cfDNA samples B12, B16 and B23 had detectable levels of H1047R mutation in untreated samples; however, enrichment of this target increased the mutant allele fraction 20–30 fold in all three cases.

### Prediction of original variant allele frequency in cfDNA and tissue samples

To estimate the fraction of mutant DNA in samples prior to enrichment, we then created a standard curve for each mutation of interest (E542K, E545K, H1047R and H1047L) with at least four known concentration samples with low VAF at 0.1, 0.5, 1, 5% (and 10% for E545K). Observed allele fraction results after enrichment were plotted against the initial mutant allele fraction and a logarithmic regression curve was fitted to the data to calculate the regression equation and coefficient of determination R^2^ for each specific assay: E542K (Fig. S3B), E545K (Fig. S3C), H1047R (Fig. S3D) and H1047L (Fig. S3E). A strong correlation was observed in all models between variant allele fraction before and after enrichment (R^2^ > 0.9). This lead to a logarithmic standard curve model being established to precisely determine initial E542K, E545K, H1047R, H1047L mutant allele fraction using a dPCR detection method.

To validate our method for low mutant allele fraction prediction from enriched samples, we used data of enriched clinical tissue biopsies (Fig. 3B) and cfDNA samples (Fig. 3C). VAF was determined by dPCR in all samples prior to enrichment and compared to the predicted VAF, calculated utilizing our novel standard curve method for enriched samples. Further, Tru-Seq panel sequencing data that was also available for tissue biopsy samples (Fig. 3D) and not cfDNA. Our proposed model predicted relatively low VAF (< 10%) in samples B03, B10 and B23), which was concordant with sequencing data for these samples. Extending this work, cfDNA samples were tested for expected mutations that had been previously detected in the matching tissue biopsy samples. *PIK3CA* E542K and E545K mutations were not detected in the B03 and B10 cfDNA samples without enrichment. However, after the enrichment, E542K was detected in B10 cfDNA sample, yet at a very low VAF of 0.04%, which was too low for accurate prediction of the initial VAF. All H1047R positive cfDNA samples B12, B16, B23 were detected by dPCR before and after enrichment. Importantly, the initial VAF observed without enrichment in each case correlated with the VAF we predicted from enriched samples (Fig. 3D).

### Validation of the VAF prediction model with blinded clinical samples

**Figure 4:**
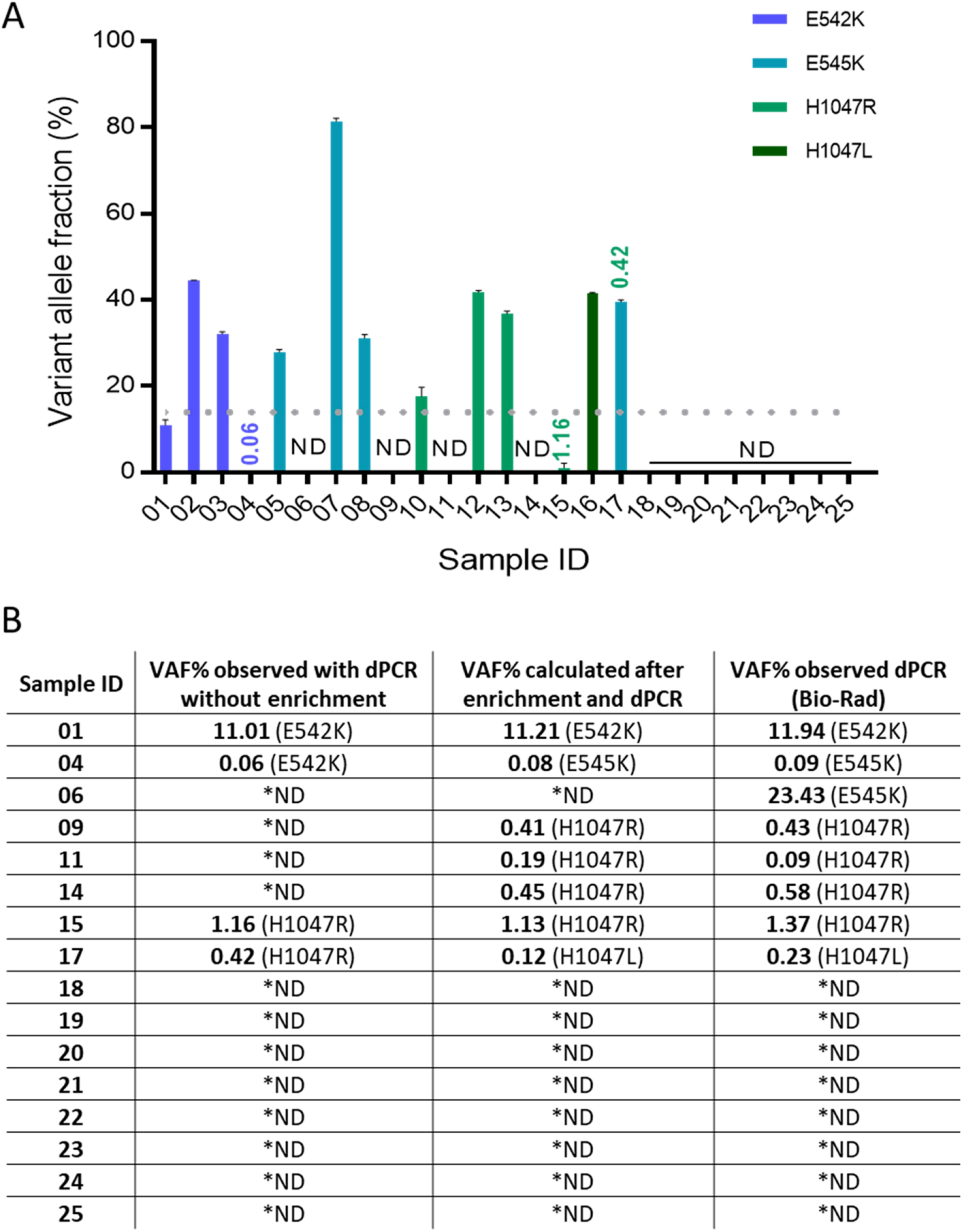
*PIK3CA* mutation detection in breast cancer FFPE biopsy tissue. (**A**) Genomic DNA isolated from FFPE tissue biopsy samples from a total of 25 breast cancer patients were subjected to dPCR analysis for *PIK3CA* E542K, E545K, H1047R and H1047L mutations. Data are shown as VAF for a detected target mutation. All samples with VAF < 15% (below the dotted line) were then enriched prior to dPCR analysis. Sample 17 was included in the exon 20 enrichment experiment due to low VAF of H1047R. The results are presented with their Poisson-based 95% confidence intervals. (**B**) The logarithmic regression method was applied to calculate initial VAF for FFPE tissue biopsy samples after enrichment with nuclease-based assays and detection with dPCR. Calculated initial VAF% results were compared to dPCR prior to enrichment and ddPCR results obtained at Institute of Cancer Research, London, UK, using a commercial Bio-Rad system and larger quantities of sample template DNA. *ND—not detected.

A blinded set of 25 breast cancer patient FFPE tissue biopsy samples was tested using our in-house triplex dPCR assays without enrichment to detect PIK3CA mutations in exon 9 (E542K, E545K) and exon 20 (H1047L, H1047R) (Fig. 4A). Samples were considered positive only if at least three droplets were positive for one of the mutations. Ten samples were identified to correctly having a high VAF (> 15%) for one of the four mutations of interest and were not included in further enrichment experiments, with the exception of sample 17, which showed a high VAF for E545K mutation and a very low VAF for H1047R mutation. Three samples were detected to have a very low mutant allele fraction (VAF < 2%) for E542K (sample 4) and H1047R (samples 15, 17). In case of sample 4, this might have been a false positive result, which is prevalent in FFPE samples due to fixation derived error^37^. Marchetti et al.^38^ suggested that fixation and embedding can result in the artefactual detection of G > A transition, which was the type of non-reproducible change we detected by dPCR analysis. Upon re-analysis of this sample, E542K (c.1624G > A) mutation was not observed. Therefore we assume this nucleotide change has been the result of paraffin embedding and fixation of the sample. The remaining 12 samples had no mutation detected (under the dotted line Fig. 4A). Therefore, in total, these 16 samples were selected for further enrichment and detection analysis.

With this selected set of samples, nuclease enrichment was applied before dPCR detection and the approximate initial VAF was calculated for each detected mutation using our established logarithmic regression model (Fig. 4B). Predicted VAF results were compared to the observed VAF in untreated samples and VAF assessed previously by Bio-Rad QX-200 digital droplet PCR platform (data from Institute of Cancer Research, London). All negative samples, 18–25, were concordant throughout the different settings. Predicted VAF in the enriched samples showed similar results to those observed externally in seven samples out of eight. Also, our proposed workflow of enrichment prior to dPCR and initial VAF prediction was shown to allow the detection of low mutant allele fraction (< 1%) in low DNA yield samples which were not detected without this step.

## Discussion

*PIK3CA* is one of the most mutated oncogenes in human malignancies and there are numerous PI3K inhibitors undergoing clinical trials. This recently resulted in the approval of Alpelisib/BYL719 for advanced PIK3CA mutation positive, hormone receptor positive, HER2 negative breast cancer patients^7^. Therefore, to identify these patients, there is a need for sensitive techniques that can detect low mutant allele fraction in high background of wild type DNA in low DNA yield and low VAF samples, such as is likely with cfDNA. In addition monitoring cancer patients for response to therapy or for disease progression requires repeated quantitative assessments of disease burden.

Here we describe a simple and robust workflow to enrich and detect common oncogenic *PIK3CA* E542K, E545K, H1047R and H1047L mutations in human genomic DNA. This includes a method to calculate the initial mutant allele frequency, which should be generally applicable to the targeted enrichment of variant sequences using nucleases. The versatility of the method has been demonstrated by combining nuclease-based enrichment with two different mutant allele detection techniques: qPCR and dPCR.

Enrichment improves the sensitivity of our described *PIK3CA* mutant allele specific qPCR assays from 5–10% to approximately 0.3–0.6%, which allows the detection of very low mutant allele fractions in clinical samples positive for one of the four most common *PIK3CA* mutations. The linear regression analysis applied to qPCR results of the enriched samples demonstrated a successful use of a standard curve to calculate the initial *PIK3CA* mutant fraction in positive DNA of tissue biopsy samples. Predicted results were comparable to the results acquired without enrichment by more sensitive techniques, such as digital PCR and next generation sequencing. Additionally, the proposed sample preparation, enrichment and qPCR data analysis workflow is low cost and simple and is accessible to any lab with qPCR capability.

*PIK3CA* mutation enrichment combined with triplex dPCR assays allowed mutation detection in tissue biopsy and cfDNA samples. By applying the logarithmic regression method, we were also able to estimate the initial mutant allele fraction in these samples. In the first set of tissue biopsies of known *PIK3CA* status, the estimated VAFs of enriched samples were 100% concordant with untreated samples tested by dPCR and Tru-Seq sequencing panel (5/5). Matching cfDNA samples showed a concordance of 80% (4/5), as enrichment of the E542K mutation enabled detection of it in sample B10. In the blinded set of 16 selected FFPE samples (with low VAF and low DNA yield), the concordance between the observed VAF without enrichment and externally observed VAF was 62.5% (10/16). It was increased to 93.75% (15/16) when we used the combination of mutation enrichment, dPCR detection and initial VAF calculation from dPCR data. Therefore, the combination of enrichment and dPCR allowed a more sensitive detection of mutations from cfDNA samples and tissue biopsy samples with low VAF. Also the ability to estimate the initial VAF from enriched samples using simple PCR analysis brings liquid biopsy closer to implementation in broad clinical setting, were quantitative measures are desired for patient follow-up testing.

A recent publication has described nuclease enrichment of mutant *PIK3CA* alleles and showed improvement in the sensitivity of ARMS detection method^39^. However, this method did not provide a quantitative measure of initial VAF prior to enrichment, which is of interest in the clinical setting to allow for patient follow-up. We aim our versatile initial VAF calculation method to be easily applied to a wide range of samples (frozen core biopsy, FFPE and cfDNA samples) subject to enrichment and to analysis by a simple qPCR method or dPCR. Although further standardisation of parameters, such as uniform DNA input, and validation in larger clinical studies are required in the future, this method provides further evidence of the potential of nuclease-based enrichment to quantify low mutant allele fractions and brings these methods closer to use at the clinical setting.

## Supplementary information

Supplementary information1

## Data Availability

Data available from the corresponding author on reasonable request.

## Abbreviations

ER: Estrogen receptor alpha
PR: Progesterone receptor
ERBB2 (HER2): Human epidermal growth factor receptor 2
PI3K: Phosphoinositide 3-kinase
cfDNA: Circulating cell-free DNA
VAF: Variant allele fraction
DSN: Duplex specific nuclease
LOD: Limit of detection
FFPE: Formalin-fixed paraffin-embedded
WBC: White blood cells
TB: Tissue biopsy
hgDNA: Human genomic DNA
