# Supplementary material for: PIK3CA mutation enrichment and quantitation from blood and tissue
Source: Sci Rep. 2020 Oct 13;10:17082. doi: 10.1038/s41598-020-74086-w (PMC7555501; doi:10.1038/s41598-020-74086-w)

**PIK3CA mutation enrichment and quantitation from blood and tissue**

Ieva Keraite^1,2^, Virginia Alvarez-Garcia^3^, Isaac Garcia-Murillas^4^, Matthew Beaney^4^, Nicholas C. Turner^4,5^, Clare Bartos^3,6^, Olga Oikonomidou^3,6^, Maïwenn Kersaudy-Kerhoas^1,2^, Nicholas R. Leslie^1*^

^1^Institute of Biological Chemistry, Biophysics and Bioengineering, Heriot-Watt University,

Edinburgh, EH14 4AS, United Kingdom

^2^Infection Medicine, Edinburgh Medical School, College of Medicine and Veterinary Medicine, The University of Edinburgh, Edinburgh, EH164SB, United Kingdom

^3^Edinburgh Cancer Research Centre, University of Edinburgh, Crewe Road South, Edinburgh, EH4 2XR, United Kingdom

^4^The Breast Cancer Now Research Centre, The Institute of Cancer Research, London, SW3 6JB, United Kingdom,

^5^Breast Unit, Royal Marsden Hospital, Fulham Road, London, SW3 6JJ, United Kingdom

^6^Edinburgh Cancer Centre, Western General Hospital, Crewe Road South, Edinburgh, EH4 2XU, United Kingdom

*All correspondence should be addressed to:

Nicholas R. Leslie, Prof.

Institute of Biological Chemistry, Biophysics and Bioengineering

School of Engineering & Physical Sciences

Heriot-Watt University

EH14 4AS Edinburgh

United Kingdom

Email: n.r.leslie@hw.ac.uk

Tel: 44-131-451-8157

**Supplementary figure legends**

**Table S1.** Clinical characteristics of the patients recruited for the study. Abbreviations: NST (no specific type), pos (positive), neg (negative), ER (estrogen receptor), PR (progesterone receptor), HER2 (human epidermal growth factor receptor 2).

**Table S2**. Primer and probe information for nuclease-based enrichment and detection with qPCR. A) Enrichment probes used for corresponding mutations. B) Primer information for mutant allele specific qPCR and the size of generated amplicons. C) Primers used for target pre-amplification.

**Table S3.** Primer and probe information for dPCR duplex and triplex assays.

**Figure S1.** Genomic DNA from cell lines containing PIK3CA E542K (A), E545K (B), H1047R (C) and H1047L (D) mutations were serially diluted in wild type DNA with decreasing mutation abundances 10, 5, 2.5, 1.25, 0.6, 0.3 and 0.15% (for H1047R and H1047L only). Mutation detection was performed in enriched samples and matched untreated controls by SYBR Green qPCR method with wild type blocking primer. (E) A linear regression equation has been estimated for data points obtained studying H1047L mutation. Data analysed by ΔΔCt method, where relative amplification was calculated in regards to WT human genomic DNA, and shown as mean mutant fold amplification ± SD. Due to a limited enrichment reaction volume (10 µl), all qPCR points were obtained in duplicates in three independent experiments (n=3). ***P < 0.001 compared with PIK3CA WT, *P < 0.05 compared with PIK3CA WT (Student’s t test using GraphPad Prism software).

**Figure S2.** Digital PCR assay result comparison with TruSeq Illumina sequenzing panel results for frozen core tissue biopsy sample DNA from breast cancer patients. Genomic DNA extracted from tissue biopsies were subjected to dPCR and targeted deep sequencing using the TruSeq Cancer Amplicon Panel (Illumina) for comparison. Data are shown as the mutant allele fraction for each sample.

**Figure S3.** A) Serial dilution of T47D mutant DNA in WT DNA to study enrichment in lower mutation fractions. Mutation detection of untreated samples was compared to enriched samples and samples following a pre-amplification step before enrichment. B) – E) . Genomic DNA from cell lines containing PIK3CA E542K (B), E545K (C), H1047R (D), and H1047L (E) mutations were serially diluted in wild type DNA with decreasing mutation abundances. Mutation detection was performed in enriched samples and matched untreated controls by dPCR. A logarithmic regression curve has been applied and equation has been estimated for every mutation.


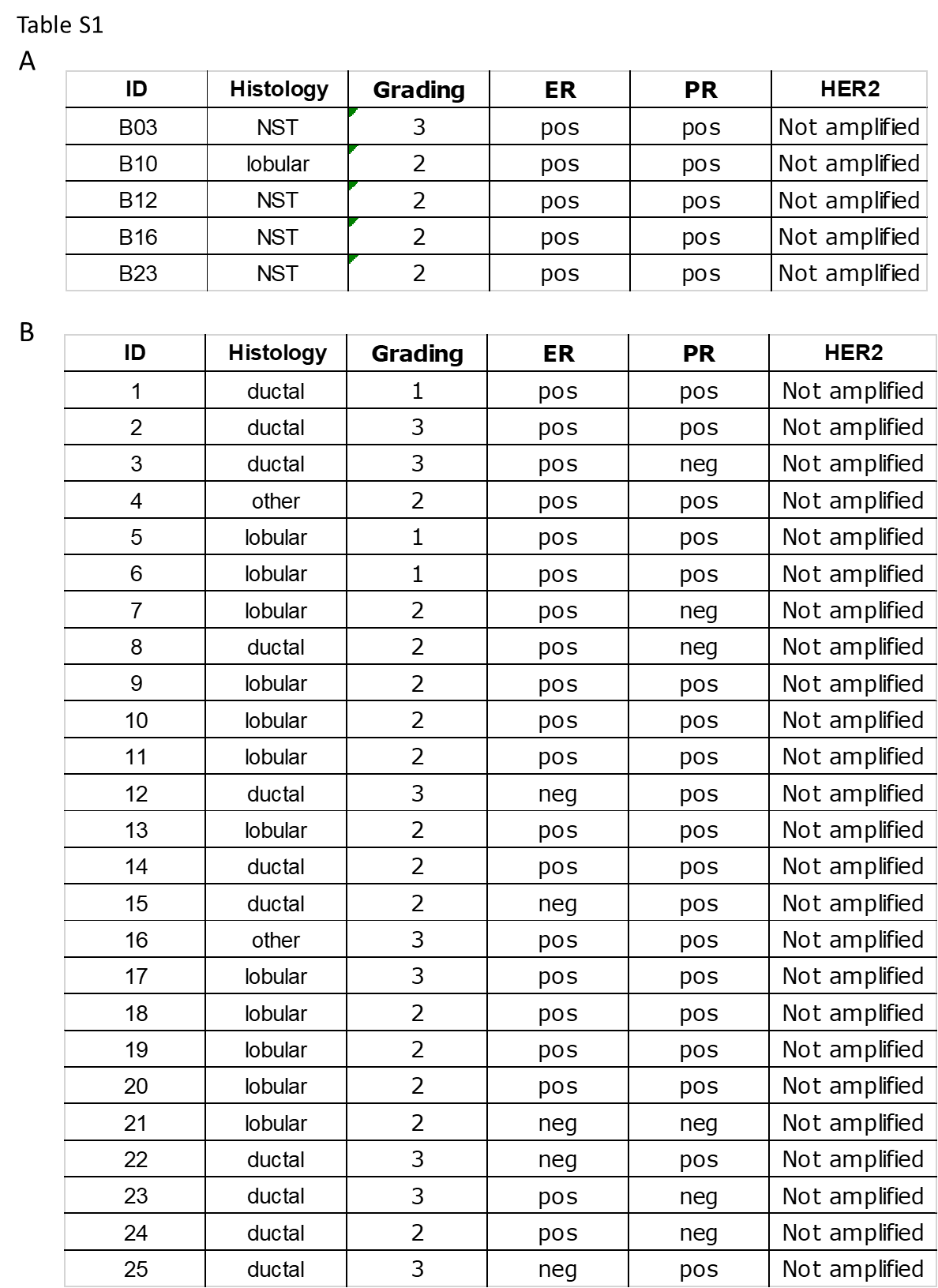


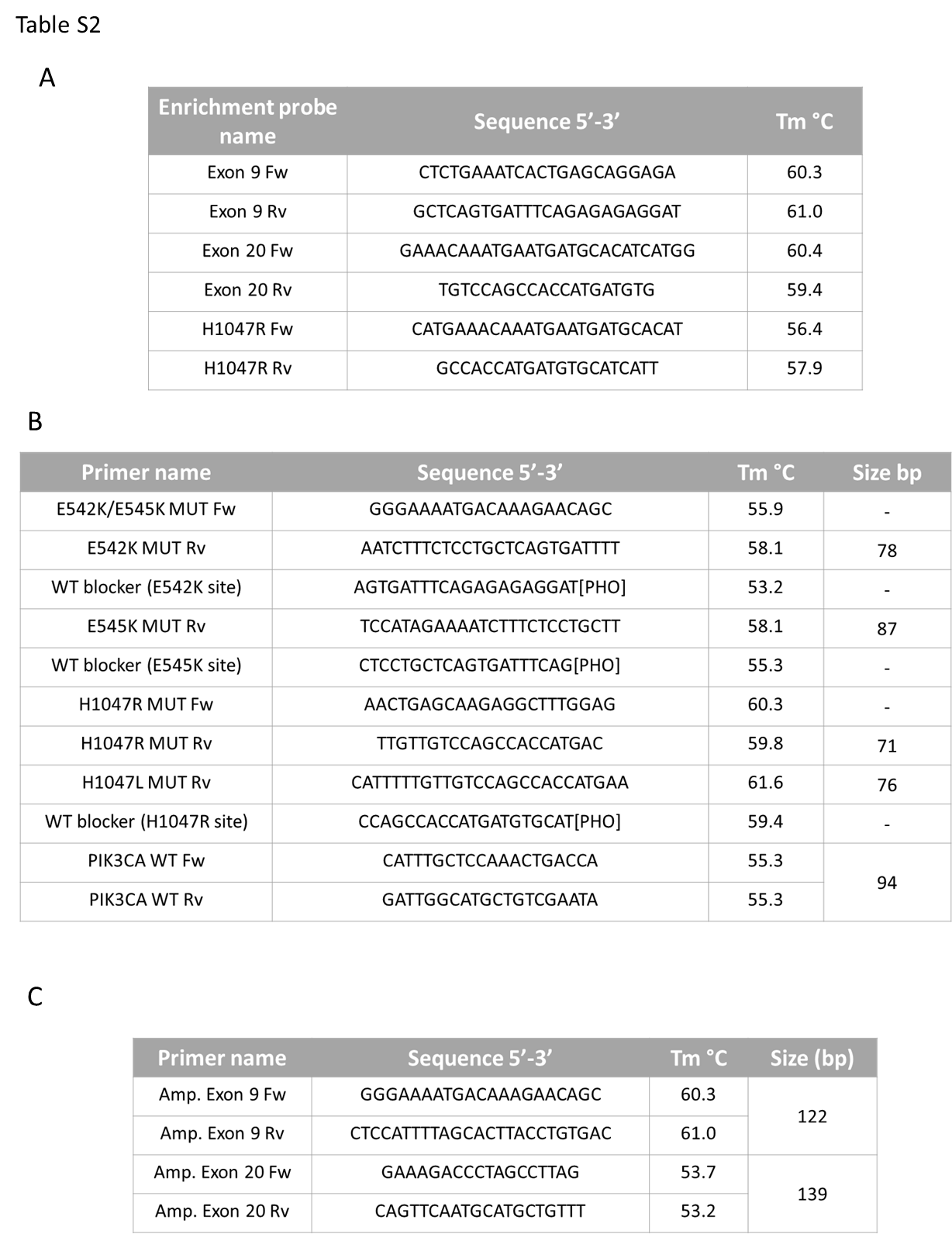


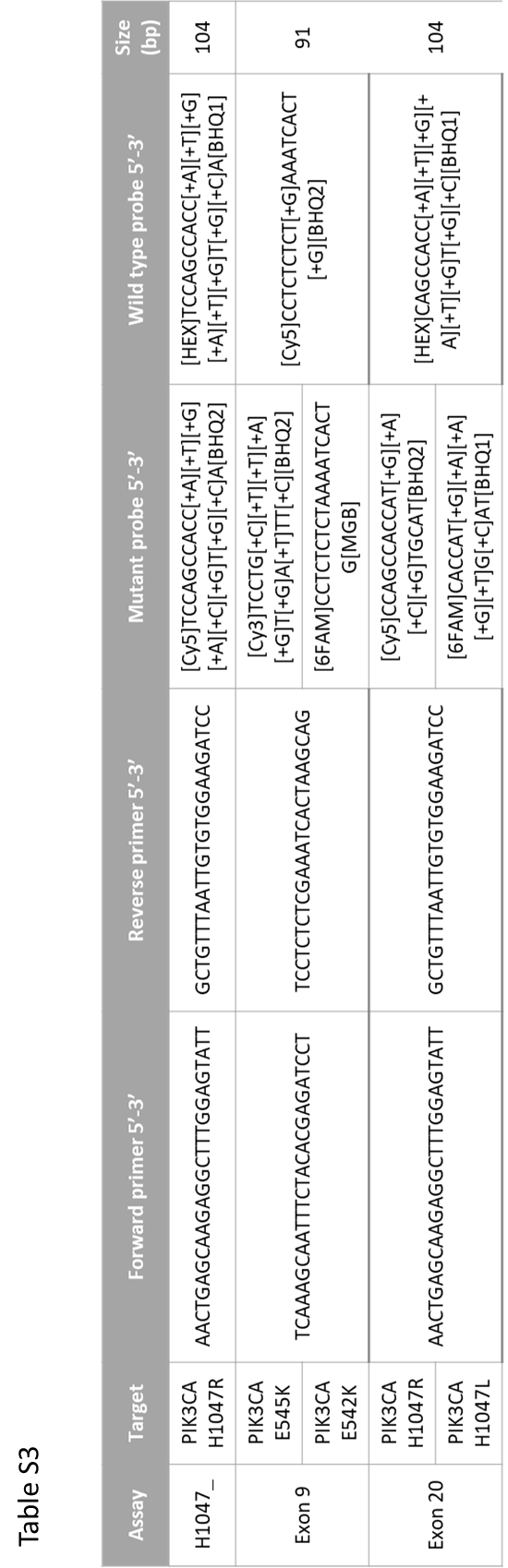


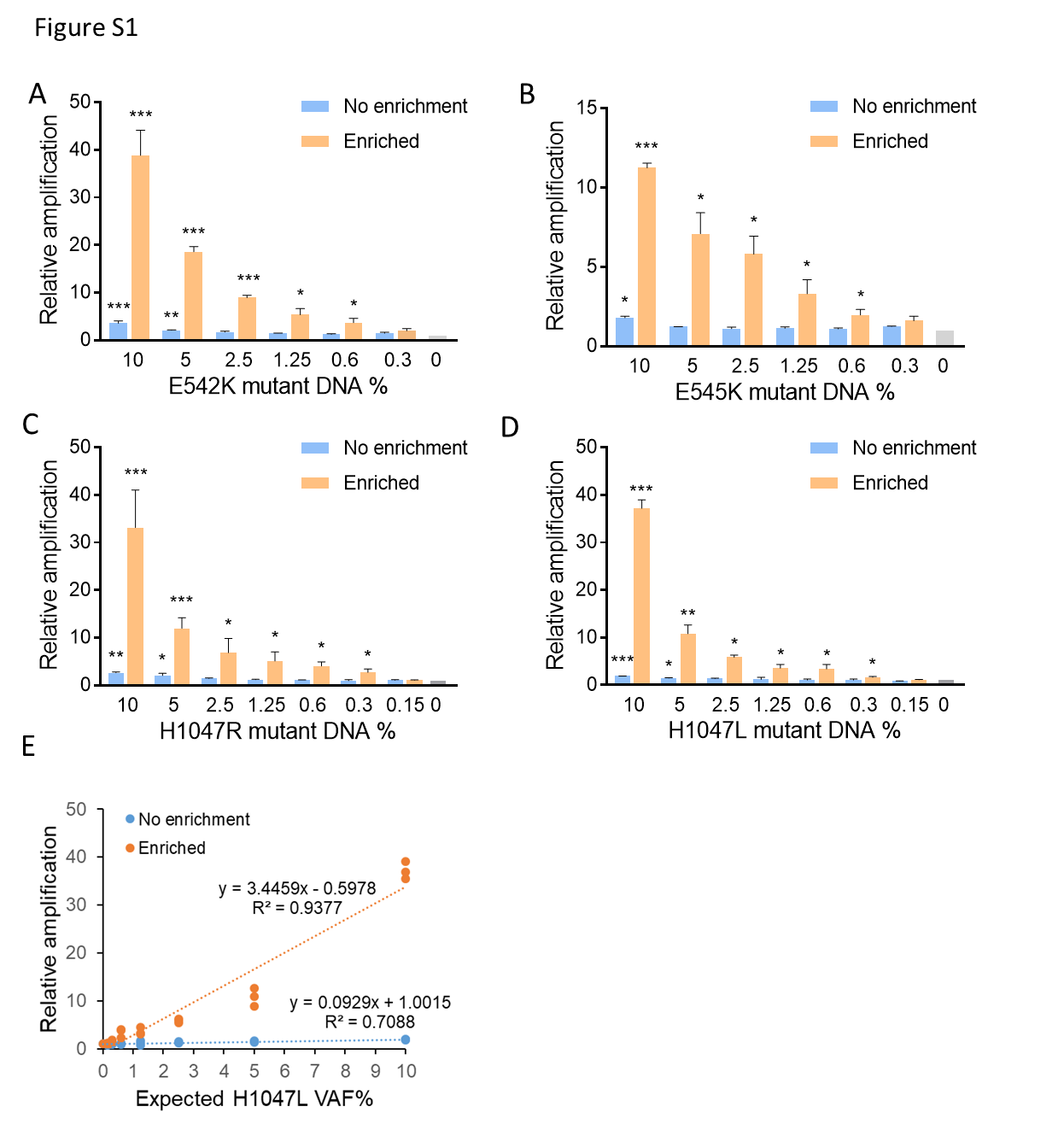


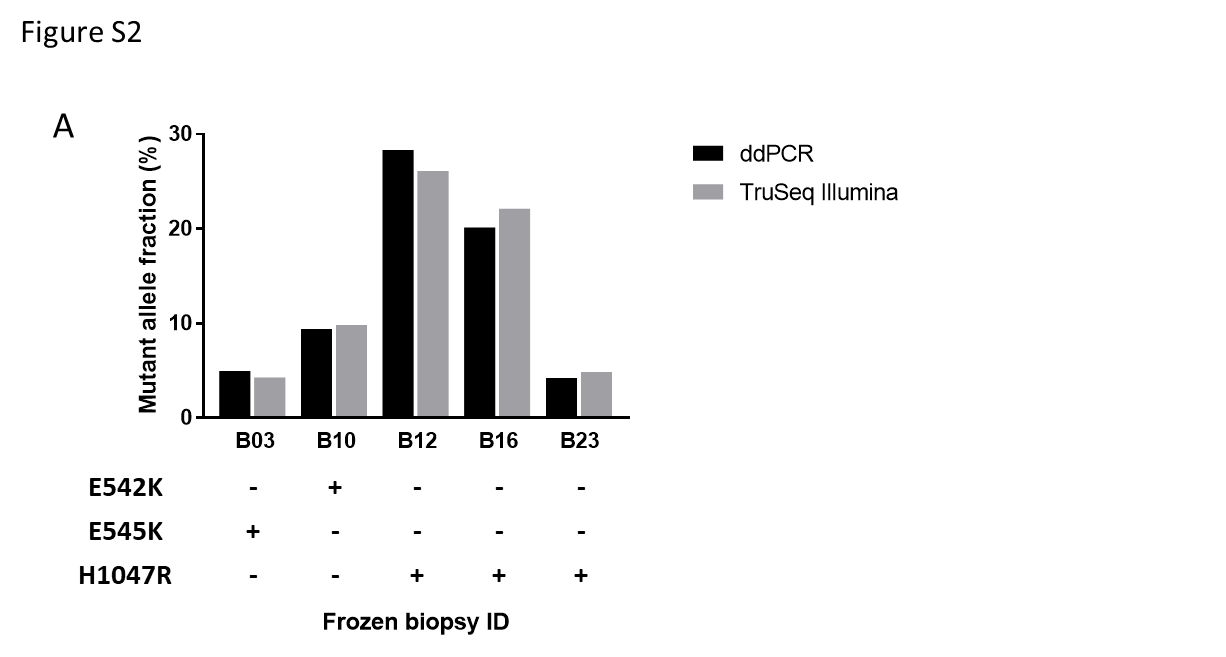


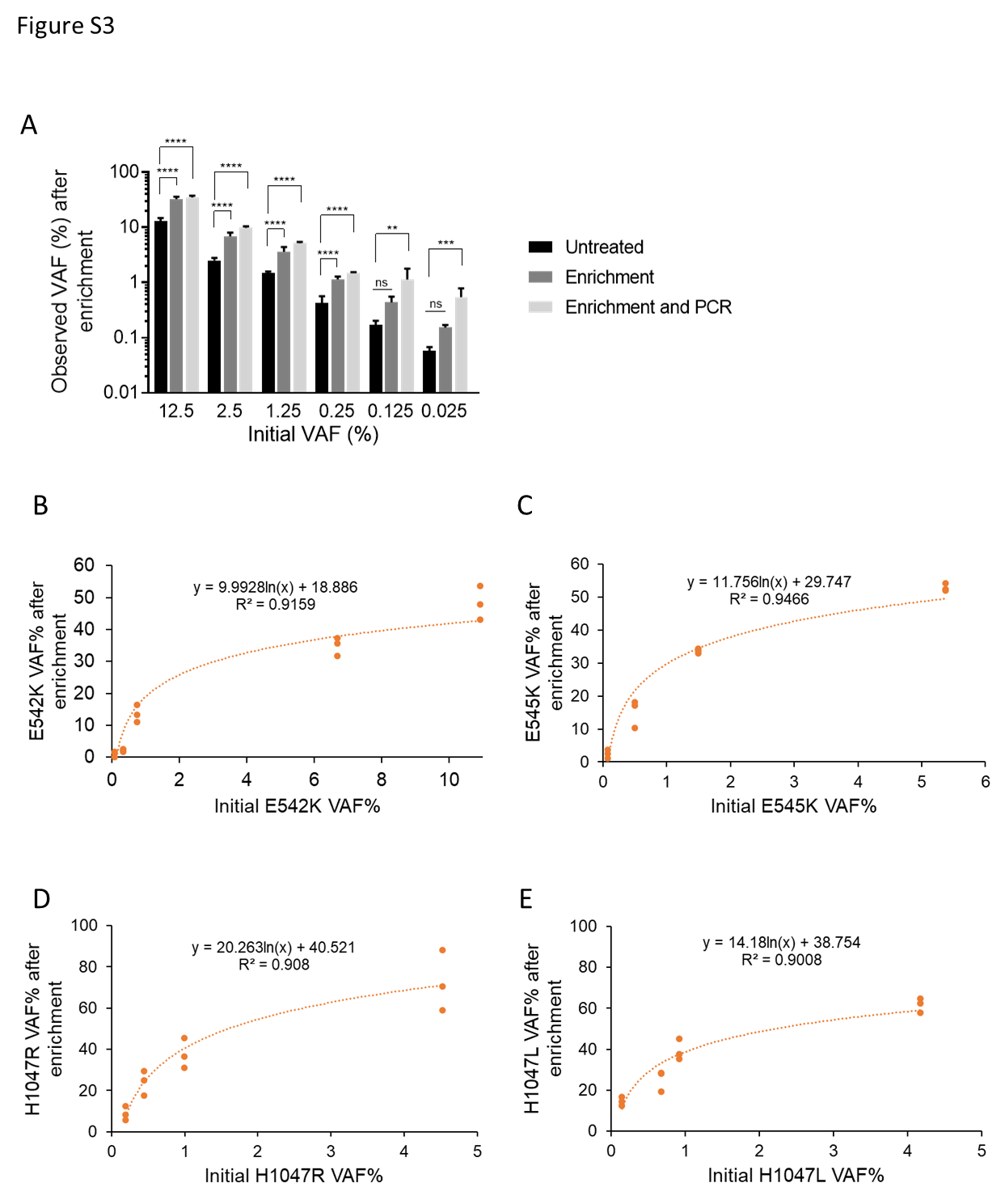

Supplement: Supplementary file 1 — Supplementary information1 [file 41598_2020_74086_MOESM1_ESM.docx]
